# Challenges for maintaining post elimination phase of visceral leishmaniasis control programme in India: A field-based study

**DOI:** 10.1371/journal.pntd.0012028

**Published:** 2024-03-07

**Authors:** Subhasish Kamal Guha, Ashif Ali Sardar, Pabitra Saha, Moytrey Chatterjee, Kingsuk Jana, Anwesha Samanta, Dipankar Maji, Prasanta Biswas, Rahul Bhattacharya, Ardhendu Kumar Maji

**Affiliations:** 1 School of Tropical Medicine, Kolkata, West Bengal, India; 2 Department of Microbiology, School of Tropical Medicine, Kolkata, West Bengal, India; 3 Department of Zoology, P. R. Thakur Govt. College, Thakurnagar, West Bengal, India; 4 Department of Health and Family Welfare, Government of West Bengal, Swasthya Bhavan, Kolkata, West Bengal, India; 5 Department of Statistics, University of Calcutta, Kolkata, West Bengal, India; Federal University of Piauí and Institute of Tropical Medicine, BRAZIL

## Abstract

**Background:**

India is going through the maintenance phase of VL elimination programme which may be threatened by the persistence of hidden parasite pools among asymptomatic leishmanial infection (ALI) and PKDL. The present work was designed to determine the burden of VL, PKDL, and ALI and to assess the role of treatment of ALI in maintaining post-elimination phase.

**Methods and finding:**

The study was undertaken in Malda district, West Bengal, India during October 2016 to September 2021. Study areas were divided into ‘Study’ and ‘Control’ arms. VL and PKDL cases of both the arms were diagnosed by three active mass surveys with an interval of one year and treated as per National guideline. ALI of ‘Study’ arm was treated like VL. ALI of ‘Control’ arm was followed up to determine their fate. Fed sand-fly pools were analysed for parasitic DNA. No significant difference was noted between the incidence of VL and PKDL in both the arms. Incidence of ALI declined sharply in ‘Study’ arm but an increasing trend was observed in ‘Control’ arm. Significantly higher rate of sero-conversion was noted in ‘Control’ arm and was found to be associated with untreated ALI burden. Parasitic DNA was detected in 22.8% ALI cases and 2.2% sand-fly pools.

**Conclusion:**

Persistence of a significant number of PKDL and ALI and ongoing transmission, as evidenced by new infection and detection of leishmanial DNA in vector sand-flies, may threaten the maintenance of post-elimination phase. Emphasis should be given for elimination of pathogen to prevent resurgence of VL epidemics.

## Author summary

The present study was conducted in a kala-azar endemic block of Malda district, West Bengal. The study areas were divided ‘Study’ and ‘Control’ arm and three active mass surveys were conducted for detection of visceral leishmaniasis (VL), post kala-azar dermal leishmaniasis (PKDL) and asymptomatic leishmanial infection (ALI). In the ‘Study’ arm, diagnosed ALI cases were treated with Liposomal Amphotericin B. No significant difference was noted between the incidence of VL and PKDL cases in ‘Study’ and ‘Control’ arm. But the incidence of ALI cases declined sharply in ‘Study’ arm and increasing trend was observed in ‘Control’ arm. A significantly higher rate of sero-conversion was noted in ‘Control’ arm. In both ‘Study’ and ‘Control’ arm, the sero-conversion rate was significantly associated with untreated ALI burden. Persistence of significant number of PKDL and ALI and ongoing transmission as evidenced by new infection and detection of leishmanial DNA in sand-flies may jeopardise the maintenance of post-elimination phase. Therefore, constant vigilance is highly needed to prevent resurgence of VL epidemic. Detection and long-term follow-up of ALI may also help to sustain the elimination. However, feasibility and cost-effectiveness study for detection and treatment of ALI should also receive attention.

## 1. Introduction

Visceral leishmaniasis (VL), also known as kala-azar, a poverty related neglected tropical disease, is caused by protozoal parasites of the genus *Leishmania* and transmitted by the *Phlebotomine* sand-fly [1]. In 1990–91, a centrally sponsored kala-azar control scheme was initiated in India resulting in sharp decline of VL cases till 1999. However, the incidence of VL again increased significantly in 2000 [2]. Accordingly, the Government of India initiated a National Health Policy in 2002 to eliminate VL by 2010 [3,4]. Thereafter, a joint VL elimination initiative supported by the World Health Organization (WHO) was launched in 2005 by three adjoining countries; India, Bangladesh, and Nepal to eliminate the disease by 2015 [3,4]. The elimination programme was aimed to reduce the annual incidence of VL below 1/10000 population at the sub-district or district level. There are four phases of VL elimination programme; a pre-control “preparatory” phase, an “attack phase” to bring down the incidence below 1/10000 populations, a “consolidation phase” of three years to keep the incidence below the target, and a “maintenance phase” to ensure the maintenance of post-elimination status beyond 2020 [5]. The initial elimination target year of 2015 was deferred to 2017, then to 2020 and to 2023 at present [6].

Nepal and Bangladesh have reached the goal in 2013 and 2016 respectively [7,8]. India is going through the attack phase of VL elimination programe and very close to achieve the elimination target except in few hotspots with ongoing transmission [9]. Epidemics of VL in India showed a cyclical pattern with an interval of 10–15 years between two peaks. The present low incidence of VL might be due to the effects of interventions of the elimination programme or due to “natural” cyclical waves or by both [10]. The present programme is targeting the elimination of the disease but not the pathogen [7]. Maintenance of post-elimination phase is thus a challenge because of persistence of hidden parasite pool among asymptomatic leishmanial infection (ALI) and undiagnosed PKDL cases.

PKDL, a cutaneous disorder, usually develops in 10–20% of treated VL cases and also among individuals having no such history [11–16]. Cross-sectional surveys revealed that a high proportion of anti-leishmanial antibody positive persons are asymptomatic [17,18]. Mathematical modelling showed that asymptomatic carriers, constituting a reservoir of parasites, may drive the epidemic in future, although their infectiousness to sandflies is not yet established [19]. Recently it was evident by Singh et al., 2021 that ALI cases were not infectious to sandflies [20]. Identification of PKDL and ALI is a challenge at community level. There is no guideline for the treatment of ALI. Several questions remain unresolved about the fate of ALI and the role of their treatment in maintenance of post-elimination phase. The present work was undertaken in kala-azar endemic areas of Malda district, West Bengal, India to detect the burden of VL, PKDL and ALI and to ascertain the role of their treatment in kala-azar elimination programme.

## 2. Materials and methods

### 2.1. Ethics statement

Before initiation of data collection, study team explained the objectives and benefits of the study to each head of the family and other members. They were also informed about non-disclosure of their identity and the liberty to withdraw from the study at any time without any explanation. Written informed consent was obtained from suspected VL, PKDL and ALI cases or from their parents/legal guardians (in case of minor) for collection of venous blood, slit skin smear (SSS) sample, and for treatment of ALI. The Ethics Committee of School of Tropical Medicine, Kolkata has approved the study protocol.

### 2.2. Study areas and study design

The study was conducted in Habibpur Block of Malda district, West Bengal during October 2016 to September 2021. Habibpur is one of the highest VL endemic blocks of Malda with an annual incidence of 3.52/10,000 population in 2014. Based on the reported VL cases of the last five years, three Gram Panchayats (GPs) (local rural administrative zone) namely Aktail, Dhumpur and Kanturka were selected and divided into ‘Study’ arm (Aktail GP) and ‘Control’ arm (Dhumpur and Kanturka GPs) (Table 1).

**Table 1:** Geographical location and population of the study arms and control arm.

	GPs	Sub-centres	Latitude (°E)	Longitude (°N)	Total population	Male (%)	Female (%)
**STUDY ARM**	AKTAIL	Kharibari	25.0405	88.3603	5518	2739 (49.6)	2779 (50.4)
		Kendpukur	25.0592	88.3314	9382	4616 (49.2)	4766 (50.8)
		Topsahar	25.0938	88.3555	4994	2382 (47.7)	2612 (52.3)
		Binodpur	25.088	88.3652	6152	3040 (49.4)	3112 (50.6)
		**TOTAL**			**26046**	**12777 (49.1)**	**13269 (50.9)**
**CONTROL ARM**	DHUMPUR	Dhumpur	24.9643	88.3057	6197	2981 (48.1)	3216 (51.9)
		Fokirakandor	24.9849	88.2935	4278	2082 (48.7)	2196 (51.3)
		Tilason	25.0131	88.3043	4733	2425 (51.2)	2308 (48.8)
		Langalbhanga	25.5218	88.3494	3635	1776 (48.9)	1859 (51.1)
		Total			18843	9264 (49.2)	9579 (50.8)
	KANTURKA	Kanturka	25.0047	88.3234	5056	2574 (50.9)	2482 (49.1)
		Laxmipur	25.0386	88.3251	3991	1927 (48.3)	2064 (51.7)
		Nimbari	25.0154	88.3664	6567	3319 (50.5)	3248 (49.5)
		Mirajpur	25.1192	88.3761	3901	1913 (49.0)	1988 (51.0)
		Total			19515	9733 (49.9)	9782 (50.1)
	**TOTAL**				**38358**	**18997 (49.5)**	**19361 (50.5)**
**GRAND TOTAL**					**64404**	**31774 (49.3)**	**32630 (50.7)**

Three active mass surveys were conducted with an interval of one year for the detection of anti-leishmanial antibody by rK39 RDT (InBios Inc., USA). The schedule of three mass surveys were: 1^st^ mass survey—during December, 2016 to May, 2017; 2^nd^ mass survey—during December, 2017 to May, 2018 and 3^rd^ mass survey–during December, 2018 to May, 2019. A 4^th^ survey was carried out after 18 months of 3^rd^ mass survey (during June, 2020 –May, 2021) to observe the fate of detected rK39 positive cases.

Before initiation of active mass survey, Auxiliary Nurse Midwife (ANMs), Accredited Social Health Activist (ASHA) workers of the selected areas were sensitized. The study team visited door-to-door for collection of demographic, socio-economic data, and information regarding history of VL/PKDL. Study team also carried out rK39 Strip test of all individuals present. All rK39 positive individuals were examined clinically and/or parasitologically. VL and PKDL cases thus diagnosed were referred to the local rural hospitals for treatment. In ‘Study’ arm, consenting ALI cases were treated with single dose of Liposomal Amphotericin B (LAmB) like VL. ALI cases of ‘Control’ arm were not treated with any antileishmanial drug but were followed up clinically and serologically at intervals of six months to ascertain disease conversion or sero-reversion.

### 2.3. Sample collection and detection of parasite or parasitic DNA

The SSS samples were collected from suspected relapsed PKDL cases for detection of parasites by microscopy and leishmanial DNA by PCR. The affected area of the skin was cleaned with 70% v/v alcohol and allowed to dry completely. The edge of the lesion was squeezed firmly between the index finger and thumb to drain the blood of the area. Using a sterile scalpel blade, a small incision was made into the dermis and blood was blotted away. The cut surface was then scraped in an outward direction to obtain the tissue fluid and cells for smear on glass slide and in NET buffer for isolation of DNA. The collected SSS smears were examined for LD bodies and *Mycobacterium sp*. following Giemsa and modified Ziehl-Neelsen (Z-N) staining respectively. Before initiation of treatment 5mL EDTA blood were drawn from the ALI cases for isolation of buffy coat. Blood fed sand-fly (*Phlebotomus argentipes*) were collected from human habitation surrounding 500m radius of active VL and PKDL patients and maintain in laboratory for 5–7 days. After that pooled (50–60 sand-fly) according to their collection site.

DNA was extracted from the SSS samples, buffy coat, and sand-fly pools by using Qiagen Blood and Tissue mini kit (Qiagen, Hilden, Germany) as per manufacturer’s instructions. Leishmania-specific nested PCR (Ln-PCR) was performed to detect Leishmanial DNA using the primers and PCR conditions described previously by Salam et al., 2010 [21]. In primary reaction the primers R221 5′-GGTTCCTTTCCTGATTTACG-3′ and R332 5′-GGCCGGTA AAGGCCGAATAG-3′) were used. For the nested PCR, 2μL of the primary PCR product was used as a template in the presence of the Leishmania-specific primers R223 (5′-TCCCATCGCAACCTCGGTT-3′) and R333 (5′-AAAGCGGGCGCGGTGCTG-3′). PCR amplification was carried out in a final volume of 50 μL that contained 10X PCR buffer, 2mM MgCl2, 0∙20 mM of each dNTP, 0∙20 μM of each primer and 1U of Taq DNA polymerase (Invitrogen). PCR conditions were initial denaturation at 94°C for 10 minutes followed by 40 cycles (35 cycle for nested reaction) each of 94°C for 30 seconds, 65°C (67°C for nested) for 30 seconds, and 72°C for 30 seconds, and a final elongation step of 72°C for 10 minutes. DNA from VL cases was taken as positive control while sterile water (Sigma-Aldrich, Gmbh) was used as negative control.

### 2.4. Case definition

As per the National Vector Borne Diseases Control Programme (NVBDCP), Government of India, case definition of different forms of leishmaniasis are as follows-

### Confirmed kala-azar case

A confirmed case of kala-azar is defined when a person from an endemic area with fever of more than 15 days duration with splenomegaly, not responding to anti-malarial and confirmed by a rapid diagnostic test (RDT) or a biopsy.

### Confirmed PKDL

A patient from a kala-azar endemic area presenting with a typically symmetrical multiple hypopigmented macules, papules, plaques or nodules with or without previous history of visceral leishmaniasis, with no loss of sensation and who is RDT positive and confirmed parasitologically by SSS or biopsy or PCR.

### Asymptomatic leishmaniasis infection (ALI)

ALI is defined as an apparently healthy individual living in kala azar endemic area and tested positive by serological test or by polymerase chain reaction or leishmanin skin test.

### Population examined

Total number of individuals examined serologically of the study areas.

### 2.5. Treatment of VL, PKDL & ALI cases

VL patients were treated with single intravenous infusion of Injection LAmB 10mg/Kg body weight and PKDL cases were treated with miltefosine for 12 weeks as per guidelines of the National Vector Borne Disease Control Program (NVBDCP) of India. In absence of any guideline for treatment, ALI cases of the study arm were treated like VL. Before initiation of treatment all confirmed VL, PKDL and ALI were screened for HIV infection.

### 2.6. Data analysis

The demographic data were entered into MS excel and crossed checked twice. The data were analysed by using free statistical software R version 4.3.2. Relative Risk (RR) with the corresponding p-value were used to compare the proportion of incidence of VL, PKDL and ALI in ‘Study’ and ‘Control’ arm. The Fisher Exact test was used to find out the association between the burden of untreated ALI and the incidence of new ALI in ‘Study’ arm and ‘Control’ arm.

## 3. Results

### 3.1. Demography of study population

The population of ‘Study’ and ‘Control’ arms was 26,046 and 38,358 respectively. Sub-centre (local rural health unit) wise population and male-female ratio are presented in Table 1. The houses were made of mud or brick; tube wells were the source of drinking water, ponds were used for household works. Cattle and pig rearing was a common practice and cattle sheds were in close vicinity of human dwellings. Most of the people were agricultural or migratory labourer.

### 3.2. Population screening

Out of a total of 64404 population, 46954 (72.9%) were examined in all three mass surveys. The number of individuals who were examined for at least once was 58066 (90.2%) (Table 2).

**Table 2:** Total population surveyed during entire project period.

Parameters	Study Arm (%)	Control Arm (%)	TOTAL (%)
**Total Population**	26046	38358	64404
**Population examined for all three active mass surveys**	19345 (74.3)	27609 (71.9)	46954 (72.9)
**Population examined for any two active mass surveys**	908 (3.5)	1433 (3.7)	2341 (3.6)
**Population examined for any one active mass survey**	1986 (7.6)	1913 (5.0)	3899 (6.1)
**Population examined for at least once**	23807 (91.4)	34259 (89.3)	58066 (90.2)
**Population not examined in any survey**	2239 (8.6)	4099 (10.7)	6338 (9.8)

In ‘Study’ arm, 20839 (79.9%), 20646 (79.3%) and 20352 (78.1%) individuals were examined during three mass surveys respectively and it was 29363 (76.5%), 29153 (76.0%) and 29090 (75.8%) in ‘Control’ arm (Table 3).

**Table 3:** Results of three active mass survey in study arm (n = 26046) and control arm (n = 38358).

Parameters	1^st^ mass survey		2^nd^ mass survey		3^rd^ mass survey	
	Study arm [n (%/proportion in 10000 population)]	Control arm [n (%/proportion in 10000 population)]	Study arm [n (%/proportion in 10000 population)]	Control arm [n (%/proportion in 10000 population)]	Study arm [n (%/proportion in 10000 population)]	Control arm [n (%/proportion in 10000 population)]
Population tested by rK39 RDTs	20839 (79.9%)	29362 (76.5%)	20646 (79.3%)	29153 (76%)	20352 (78.1%)	29090 (75.8%)
Total rk39 positive	658 (315.8/10000 population)	889 (302.8/10000 population)	565 (273.7/10000 population)	914 (313.5/10000 population)	395 (194.1/10000 population)	746 (256.4/10000 population)
rK39 positive with past history of VL/PKDL	495 (237.5/10000 population)	611 (208.1/10000 population)	396 (191.8/10000 population)	581 (199.3/10000 population)	271 (133.2/10000 population)	405 (139.2/10000 population)
rK39 positive without past history of VL/PKDL	163 (78.2/10000 population)	278 (94.7/10000 population)	169 (81.9/10000 population)	333 (114.2/10000 population)	124 (60.9/10000 population)	341 (117.2/10000 population)
Diagnosed as VL	3 (1.4/10000 population)	7 (2.4/10000 population)	2 (0.97/10000 population)	4 (1.4/10000 population)	3 (1.5/10000 population)	6 (2.1/10000 population)
Diagnosed as PKDL	17 (8.2/10000 population)	13 (4.4/10000 population)	4 (1.9/10000 population)	5 (1.7/10000 population)	2 (0.98/10000 population)	11 (3.8/10000 population)
Untreated ALI from 1^st^ / 2^nd^ survey	-	-	40 (55.6%)	162 (59.4%)	39 (56.5%)	196 (60.1%)
New ALI from negative in 1^st^ /2^nd^ survey	-	-	31 (15.0/10000 population)	103 (35.3/10000 population)	14 (6.9/10000 population)	111 (38.2/10000 population)
New ALI from test not done in 1^st^ / 2^nd^ survey	-	-	23 (11.1/10000 population)	60 (20.6/10000 population)	7 (3.4/10000 population)	22 (7.6/10000 population)
Total ALI	158 (75.8/10000 population)	271 (92.3/10000 population)	94 (45.5/10000 population)	325 (111.5/10000 population)	60 (29.5/10000 population)	329 (113.1/10000 population)
ALI treated	86 (54.4%)	Treatment not given	25 (26.6%)	Treatment not given	-	Treatment not given
ALI untreated	72 (45.6%)	271 (100%)	69 (73.4%)	325 (100%)	-	-

### 3.3. Incidence of total rK39 positive cases in ‘Study’ and ‘Control’ arm

In ‘Study’ arm, 658, 565 and 395 rK39 positive cases with a proportion of 315.8, 273.7 and 194.1 per 10000 populations were detected during three mass surveys respectively. The number and proportion of such cases in ‘Control’ arm were 889 (302.8), 914 (313.5) and 746 (256.4) (Table 3). The proportion of rK39 positive cases showed a declining trend in ‘Study’ arm but an increasing trend in ‘Control’ arm.

### 3.4. Incidence of VL in ‘Study’ and ‘Control’ arm

In ‘Study’ arm, VL was diagnosed in 3, 2 and 3 cases with a proportion of 1.4, 0.97 and 1.5 per 10000 populations during three mass surveys respectively. It was 7, 4 and 6 cases with a proportion of 2.4, 1.4 and 1.7 in ‘Control’ arm (Table 3). The target of the VL elimination programme i.e., incidence of VL<1/10000 population was recorded only during 2^nd^ year in ‘Study’ arm. No significant difference was noted between the incidence of VL cases in ‘Study’ and ‘Control’ arms during 1^st^ (RR = 0.604, p = 0.465), 2^nd^ (RR = 0.706, p = 0.688) and 3^rd^ (RR = 0.715, p = 0.635) survey.

### 3.5. Incidence of PKDL in ‘Study’ and ‘Control’ arm

In ‘Study’ arm, 17, 4 and 2 PKDL cases with a proportion of 8.2, 1.9 and 0.98 per 10000 populations were diagnosed during three mass surveys. It was 13, 5 and 11 with a proportion of 4.4, 1.7 and 3.8 in ‘Control’ arm (Table 3). The observed incidence of PKDL in both the arms were not significantly different during 1^st^ (RR = 1.843, p = 0.097), 2^nd^ (RR = 1.13, p = 0.856) and 3^rd^ (RR = 0.259, p = 0.079) survey. Apart from this, 8 more PKDL cases (1 in ‘Study’ and 7 in ‘Control’ arm) were diagnosed during 4^th^ survey. Out of 60 diagnosed PKDL, 3 had no previous history of VL.

### 3.6. Incidence of ALI in ‘Study’ and ‘Control’ arm

In ‘Study’ arm, 158, 54 and 21 ALI were detected during 1^st^, 2^nd^ and 3^rd^ mass surveys respectively. It was 271, 164 and 133 in ‘Control’ arm (Table 3). The total number of ALI declined sharply in ‘Study’ arm while it increased in ‘Control’ arm. Although mere significant difference in incidence of ALI was noted during 1^st^ (RR = 0.821, p = 0.050) survey, highly significant differences were observed during the 2^nd^ (RR = 0.408, p < 0.0001) and 3rd (RR = 0.261, p < 0.0001) survey.

### 3.7. Detection of parasitic DNA among suspected PKDL, ALI and sand-fly pool by PCR

Out of 58 SSS samples collected from suspected PKDL patients, 43 (74.1%) were positive for LD bodies and/or parasitic DNA. Of which, 39 were positive for both LD bodies and PCR, 3 for PCR only. Interestingly, 1 was positive for LD bodies but negative for PCR. A total of 114 blood samples were collected from ALI, among which 26 (22.8%) samples were positive for leishmanial DNA. Leishmanial DNA was detected in 3 (2.2%) sand fly pools, out of 138 pools collected and examined.

### 3.8. Treatment of VL, PKDL and ALI

A total of 25 VL and 60 PKDL were diagnosed from ‘Study’ and ‘Control’ arms and treated successfully with LAmB and miltefosine respectively. In ‘Study’ arm, out of 233 diagnosed ALI, only 111 consented for treatment. Before treatment initiation, all patients were screened for HIV. But none of them came as HIV reactive. The demography of all treated VL, PKDL and ALI are given in Table 4. The adverse effects encountered during LAmB infusion include respiratory distress (n = 6), nausea and vomiting (n = 2), backache (n = 15), urticaria (n = 1), chest pain (n = 1), dizziness (n = 1) and sweating (n = 3). All adverse drug reaction were mild in nature and managed symptomatically. All could be discharged on the same day of infusion. None had recurrent and delayed side effects of LAmB.

**Table 4:** Demography of treated ALI, VL, and PKDL patients.

Characteristics	ALI cases (n = 111)	VL cases (n = 25)	PKDL cases (n = 60)
**Age (years)**			
Mean ± SD Range SEM	40.68± 14.57 3–70 1.38	28.38± 15.42 4.5–65 3.08	25.67± 13.19 1–65 1.70
**Sex (%)**			
Male Female	57 (51.35%) 54 (48.65%)	14 (56%) 11 (44%)	32 (53.33%) 28 (46.67%)
**Body weight (Kg)**			
Mean ± SD Range SEM	46.7± 11.8 10–74 1.12	49.92± 14.79 13–69 2.96	51.48± 13.32 5–78 1.72

### 3.9. Pattern of sero-reversion, sero-conversion and disease transformation in ‘Study’ and ‘Control’ arm

**Fig 1:**
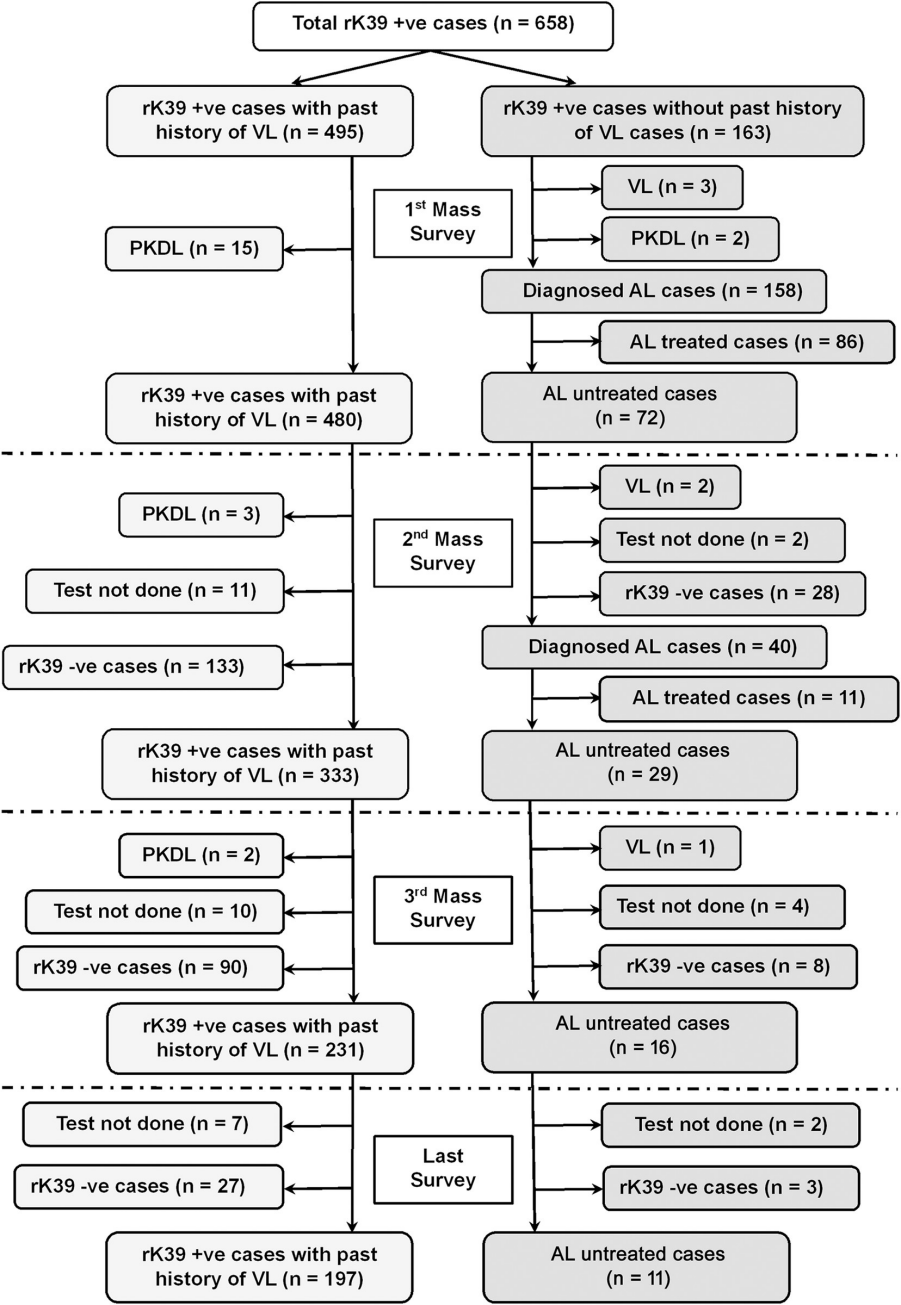
Follow-up of rK39 positive cases with and without past history of VL in study arm.

**Fig 2:**
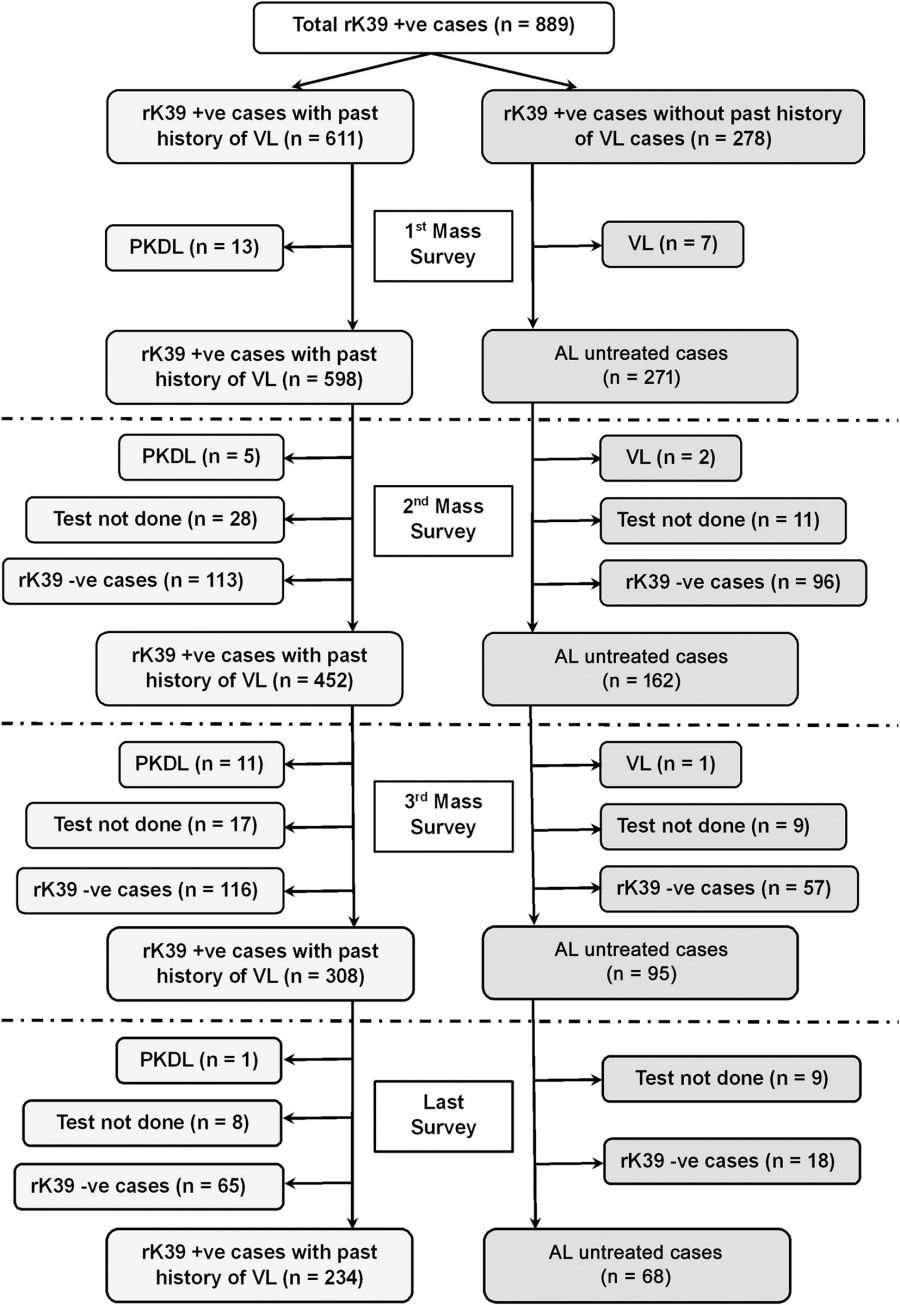
Follow-up of rK39 positive cases with and without past history of VL in control arm.

**Fig 3:**
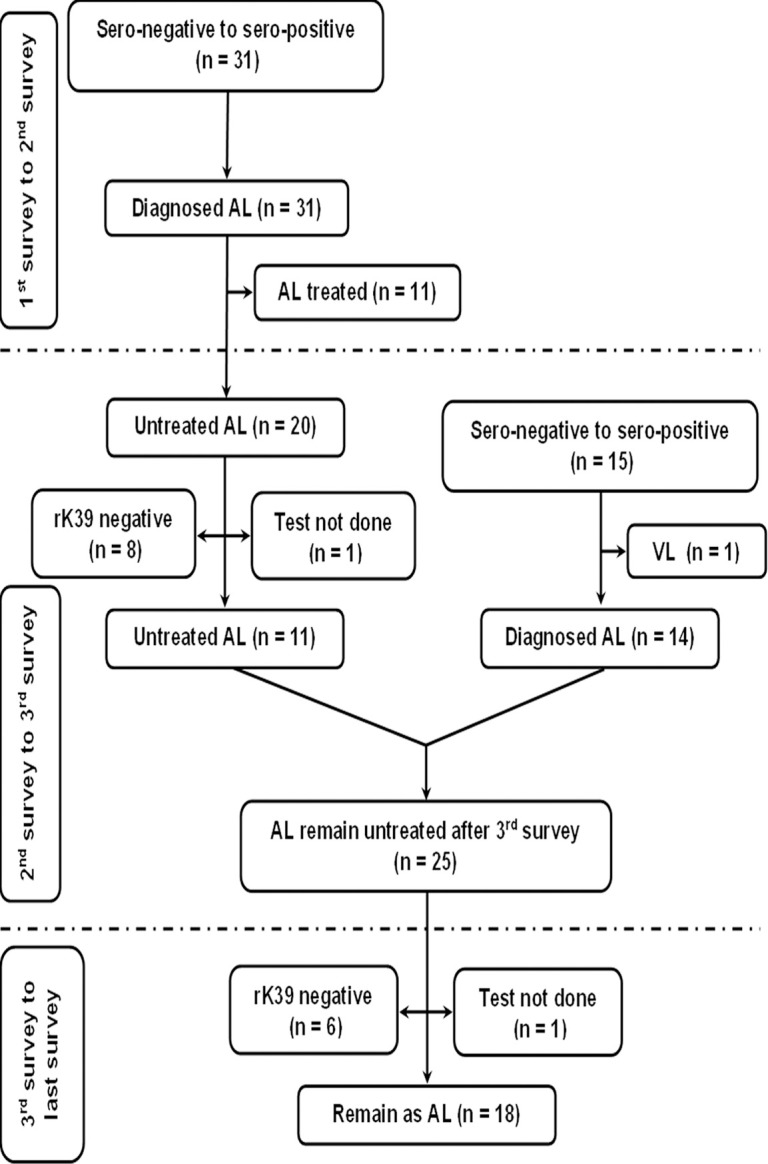
Follow up of new infection (sero-negative to sero-positive) between 1^st^ and 2^nd^ mass survey and 2^nd^ and 3^rd^ mass survey in the study arm.

**Fig 4:**
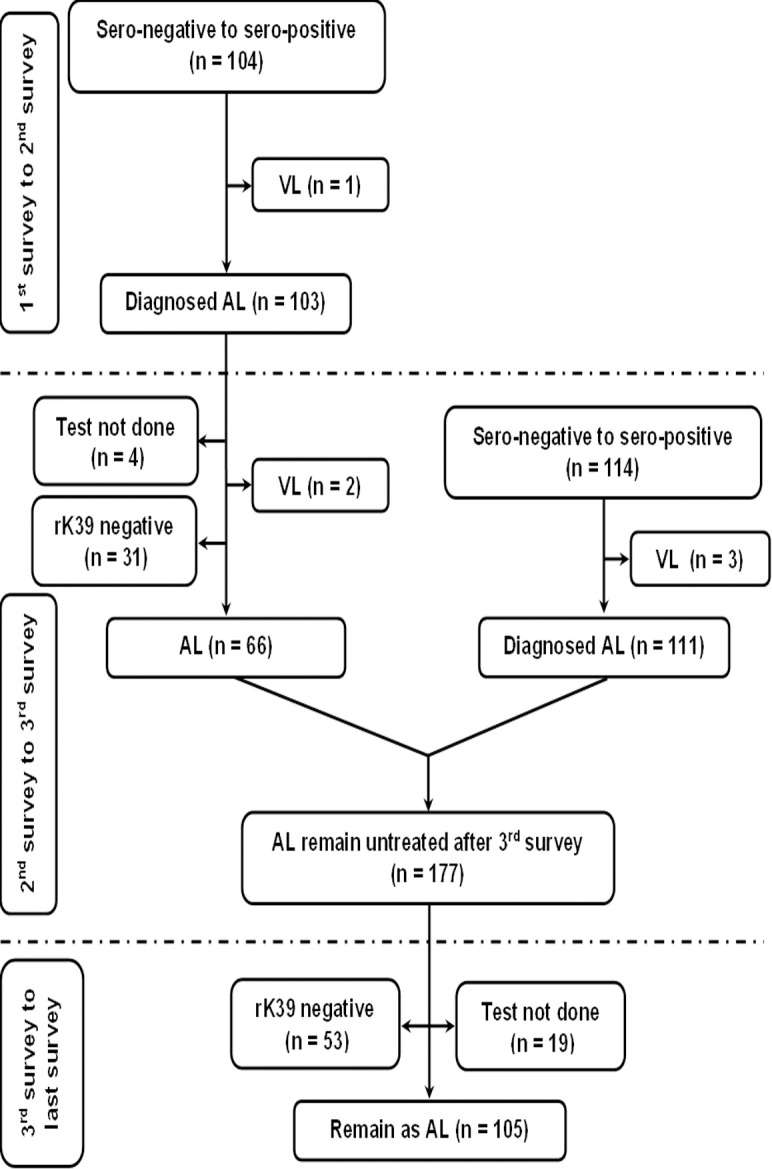
Follow up of new infection (sero-negative to sero-positive) between 1^st^ and 2^nd^ mass survey and 2^nd^ and 3^rd^ mass survey in the control arm.

**Fig 5:**
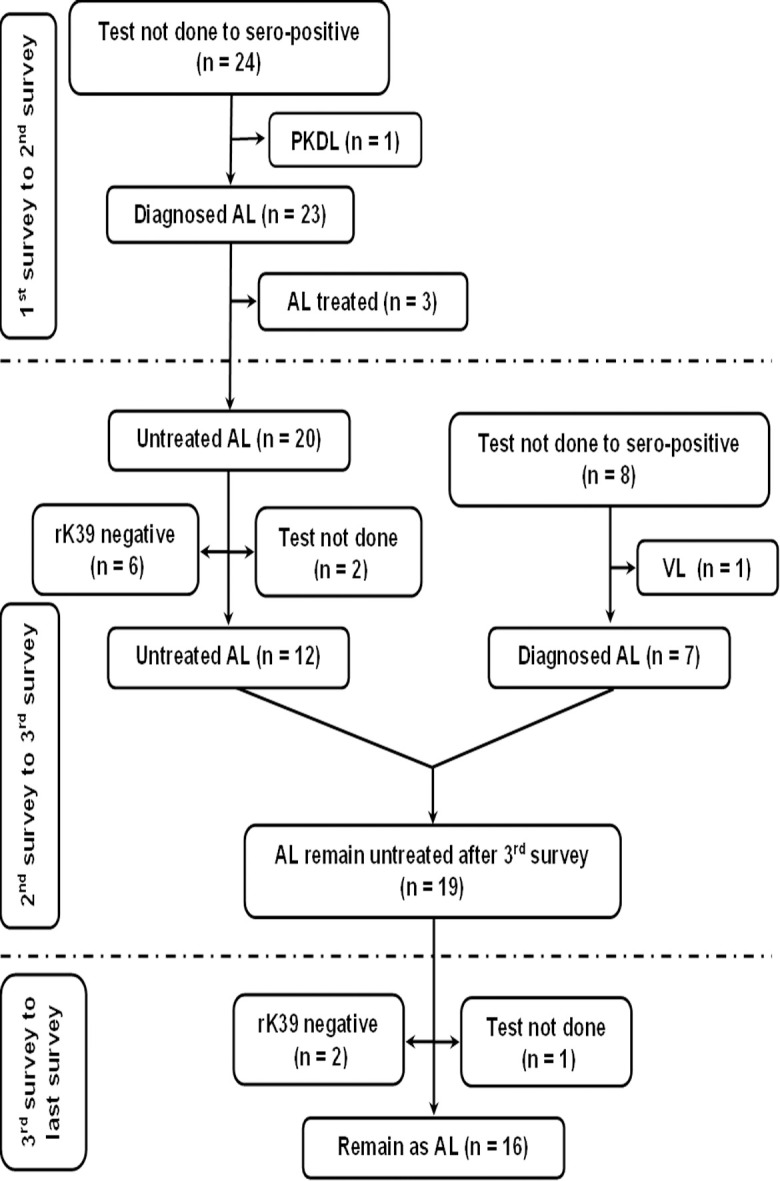
Follow up of test not done to rK39 positive between 1^st^ and 2^nd^ mass survey and 2^nd^ and 3^rd^ mass survey in the study arm.

**Fig 6:**
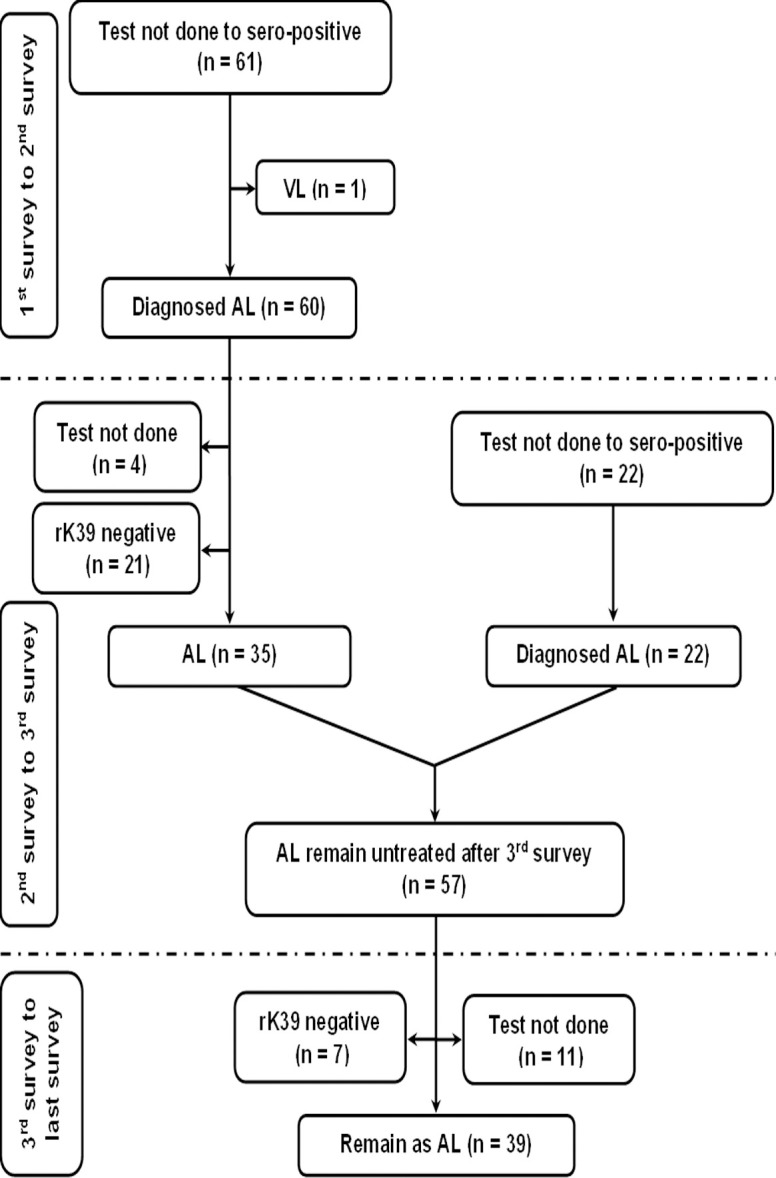
Follow up of test not done to rK39 positive between 1^st^ and 2^nd^ mass survey and 2^nd^ and 3^rd^ mass survey in the control arm.

The fate of rK39 positive subjects with or without past history of VL/PKDL is presented in Figs 1 and 2. The overall rate of sero-reversion was 32.45% and 33.33% in ‘Study’ and ‘Control’ arm respectively (Table 5). No significant difference was noted in terms of sero-reversion between two arms (p = 0.947). Disease conversion i.e., transformation of ALI into clinical VL were recorded in 2 (2.86%) and 1 (1.61%) individual between 1^st^ & 2^nd^ and 2^nd^ & 3^rd^ mass survey respectively in ‘Study’ arm. In ‘Control’ arm, it was 2 (0.76%) and 3 (0.97%). The overall disease conversion rate was similar in ‘Study’ and ‘Control’ arms (2.27% vs. 0.88%) without any significant differences (p = 0.176) (Table 5). The pattern of sero-conversion i.e., from rK39 negative to rK39 positive in ‘Study’ and ‘Control’ arms are presented in Figs 3 and 4. In ‘Study’ arm, the rate of sero-conversion per 10000 populations was 15.36 and 7.46 between 1^st^ & 2^nd^ and 2^nd^ & 3^rd^ mass survey, respectively. It was 36.52 and 40.36 per 10000 population during same period in ‘Control’ arm. A significantly higher rate of sero-conversion was noted in ‘Control’ arm between 1^st^ and 2^nd^ (p<0.0001) and 2^nd^ and 3^rd^ mass survey (p<0.0001). Fisher exact test revealed that the sero-conversion rate was significantly associated with the untreated ALI burden during 1^st^ and 2^nd^ mass survey (p<0.00001) and between 2^nd^ and 3^rd^ mass survey (p<0.00001) in both ‘Study’ arm and ‘Control’ arm. The fate of test not done to rK39 positive cases between 1^st^ & 2^nd^ and 2^nd^ & 3^rd^ survey in ‘Study’ and ‘Control’ arm are presented in Figs 5 and 6.

**Table 5:** Pattern of sero-reversion, disease conversion and sero-conversion in study and control arm.

Fate	Particulars	Study arm	Control arm
**SERO-REVERSION**	No. ALI diagnosed in 1^st^ survey	158	271
	No. of ALI treated	86	-
	No. of ALI untreated	72	271
	No. of ALI converted into VL during 1^st^ and 2^nd^ survey	2	2
	No. of ALI not tested during 2^nd^ survey	2	11
	No. of ALI became -ve between 1^st^ and 2^nd^ survey	28	96
	**Rate of sero-reversion during 1**^**st**^ **and 2**^**nd**^ **survey**	**40.0% (28/70)**	**36.92% (96/260)**
	No. cumulative ALI cases after 2^nd^ survey	94	325
	No. of ALI treated	25	-
	No. of ALI untreated	69	325
	No. of ALI converted into VL during 1^st^ and 2^nd^ survey	1	2
	No. of ALI not tested during 2^nd^ survey	7	17
	No. of ALI became -ve between 2^nd^ and 3^rd^ survey	22	109
	**Rate of sero-reversion during 2**^**nd**^ **and 3**^**rd**^ **survey**	**35.48% (22/62)**	**35.38% (109/308)**
	No. cumulative ALI cases after 3^rd^ survey	60	328
	No. of ALI untreated	60	328
	No. of ALI converted into VL during 1^st^ and 2^nd^ survey	0	0
	No. of ALI not tested during 2^nd^ survey	4	35
	No. of ALI became -ve between 3^rd^ and last survey	11	82
	**Rate of sero-reversion during 3**^**rd**^ **and last survey**	**19.64% (11/56)**	**27.98% (82/293)**
	**Overall rate of sero-reversion during study period**	**32.45% (61/188)**	**33.33% (287/861)**
**SERO-CONVERSION**	No. rK39 -ve individuals tested in 1^st^ survey	20181	28473
	No. rK39 -ve individuals became +ve in 2^nd^ survey	31	104
	**Rate of sero-conversion between 1**^**st**^ **& 2**^**nd**^ **survey/10000 population**	**15.36 (31/20181)**	**36.53 (104/28473)**
	No. rK39 -ve individuals tested in 2^nd^ survey	20081	28239
	No. rK39 -ve individuals became +ve in 3^rd^ survey	15	114
	**Rate of sero-conversion between 2**^**nd**^ **& 3**^**rd**^ **survey /10000 population**	**7.46 (15/20081)**	**40.36 (114/28239)**
**DISEASE CONVERSION**	No. ALI diagnosed in 1^st^ survey	158	271
	No. of ALI untreated	72	271
	No. of ALI not tested during 2^nd^ survey	2	11
	No. of ALI converted to VL between 1^st^ and 2^nd^ survey	2	2
	**Rate of disease conversion during 1**^**st**^ **and 2**^**nd**^ **survey**	**2.86% (2/70)**	**0.76% (2/260)**
	No. cumulative ALI cases after 2^nd^ survey	94	325
	No. of ALI untreated	69	325
	No. of ALI not tested during 2^nd^ survey	7	17
	No. of ALI converted to VL between 2^nd^ and 3^rd^ survey	1	3
	**Rate of disease conversion during 2**^**nd**^ **and 3**^**rd**^ **survey**	**1.61% (1/62)**	**0.97% (3/308)**
	**Overall rate of disease conversion during study period**	**2.27% (3/132)**	**0.88% (5/568)**

## 4. Discussion

Maintenance of post elimination phase is challenging due to persistence of hidden parasite pool among ALI and PKDL. In the present study the burden of VL, ALI and PKDL were determined. VL and PKDL were treated immediately. The fate of ALI i.e., sero-reversion, disease conversion and pattern of sero-conversion were ascertained. Role of treatment of ALI cases for elimination and disease transmission was also assessed.

In the present study, a total of 800 ALI and 25 VL cases were diagnosed with a ratio of 32:1. A higher ratio of ALI vs. VL was reported from Spain (50:1) [22]. In contrast much lower ALI: VL ratio was reported from Indian subcontinent (4–17:1), Sudan (2.4:1), Kenya (4:1) and Ethiopia (5.6:1) [22]. The overall (both in ‘Study’ and ‘Control’ arm) sero-reversion i.e., sero-positive to sero-negative was recorded in 37.58%, 35.41% and 26.93% between 1^st^ & 2^nd^, 2^nd^ & 3^rd^ and 3^rd^—last survey respectively. Similar observation was reported from different parts of India and abroad [23–26]. The overall (both in ‘Study’ and ‘Control’ arm) rate of disease conversion i.e., ALI to VL was recorded among 1.21% and 1.08% during 1^st^ and 2^nd^ year of study respectively. The overall rate of disease conversion among ALI was 1.14%. Such conversion rate varied from 1.5–23% as reported from different endemic areas [17,24,27–30]. Importantly, 256 cases remained as ALI even after five years of follow-up. Similar observation was also recorded among 164 ALI following 4 years of diagnosis [31]. In ‘Study’ arm, new infection i.e., sero-negative to sero-positive was observed in 15.01 and 6.87 per 10000 population between 1^st^ & 2^nd^ and 2^nd^ & 3^rd^ survey. A significantly higher rate of sero-conversion was observed in ‘Control’ arm during the same period. A significant association was recorded between new infection (sero-conversion) and burden of untreated ALI in both ‘Study’ and ‘Control’ arm (p < 0.00001).

The only additional intervention i.e., treatment of ALI was applied in ‘Study’ arm. Subjects with ALI were apparently healthy and a significant proportion of them refused to accept the treatment. The role of ALI in disease transmission is yet to be well established [20]. In the present study, the detection of leishmanial DNA in 22.8% ALI may help to enlighten it. It may be investigated and confirmed by xenodiagnosis studies. Though, no guideline is available for the treatment of ALI but an attempt was made to treat such cases as a pilot initiative to evaluate the role of ALI treatment in disease elimination and transmission reduction. During first three years of the study, the proportion of VL incidence constantly remained above 1 per 10000 populations (target of VL elimination programme) except in 2^nd^ year of the ‘Study’ arm. Interestingly, disease conversion from ALI was recorded in 2 and 1 cases in ‘Study’ arm and 2 and 3 cases in ‘Control’ arm during 2^nd^ and 3^rd^ year of the study. Considering the potential side-effects of LAmB, unwillingness of apparently healthy ALI subjects to receive LAmB treatment and unavailability of user-friendly methods to diagnose ALI, the treatment of such cases may be avoided. Instead, they should be followed up for a long period for development of VL/PKDL. Similar recommendation was also made by Chakravarty et al., 2019 from a field-based study in Bihar, India [32]. Considering the flight range of sand-fly, ALI may be detected by active mass screening surrounding 500 meters radius of active or recently treated VL and PKDL patients and followed up for a long period by community health workers.

PKDL is another challenge for VL elimination programme and also for prevention of resurgence following disease elimination. PKDL patients are apparently healthy without any systemic involvement [33]. Poor people do not bother about initial skin lesions and do not seek treatment, thus maintaining the parasite in the community. Xenodiagnosis studies showed that all forms of PKDL are infectious to sandfly [34–36]. Addy and Nandy (1992) reported that a single PKDL case was capable of initiating VL epidemic in favourable conditions [37]. So, prompt and proper management of PKDL is essential for maintaining the post-elimination phase. In the present study, PKDL cases were diagnosed in all four surveys from both ‘Study’ and ‘Control’ arms. The diagnosis of PKDL is often difficult due to late treatment seeking behaviour of the patients, close resemblance to many common skin diseases, and unavailability of parasitological diagnostics at community level which delay the early diagnosis and prompt treatment. Active door-to-door mass survey at a regular interval of six months is helpful for diagnosis and management of such cases. Since the present modalities of VL elimination programme of India focuses on VL only, such PKDL and ALI remain in the community and serve as reservoir of pathogens for disease transmission. It is reflected by the incidence of a significant number of new ALI and detection of parasitic DNA among fed sand-fly pool indicating ongoing transmission in the community.

Persistence of significant number of PKDL and ALI and ongoing transmission as evidenced by new infection and detection of leishmanial DNA in sand-flies may jeopardise the maintenance of post-elimination phase. Constant vigilance is highly needed to prevent resurgence of VL epidemic. Thrust should be given for elimination of pathogens by follow-up of previously treated VL and PKDL cases at regular interval by active case search. Detection and long-term follow-up of ALI may also help to sustain the elimination. However, feasibility and cost-effectiveness study for detection and treatment of ALI should also receive attention.
